# Molecular therapy for the treatment of hepatocellular carcinoma

**DOI:** 10.1038/sj.bjc.6604784

**Published:** 2008-11-18

**Authors:** T F Greten, F Korangy, M P Manns, N P Malek

**Affiliations:** 1 Department of Gastroenterology, Hepatology and Endocrinology, Center for Internal Medicine, Medical School of Hannover, Hannover, Germany

**Keywords:** molecular therapy, receptor tyrosine kinase inhibitors, sorafenib, EGFR, vascular targeting agent

## Abstract

Hepatocellular carcinoma (HCC) is the fifth most common cancer worldwide. Conventional cytotoxic chemotherapy has failed to show a substantial benefit for patients with HCC. Recently, a number of new drugs targeting molecular mechanisms involved in liver cell transformation have entered into clinical trials and led to encouraging results. In this review we summarise this data and point to a number of new compounds, which are currently being tested and can potentially broaden our therapeutic arsenal even further.

Hepatocellular carcinoma is the third most common cause of cancer-related death worldwide with 600 000 patients dying of this disease every year (Parkin *et al*, 2005). Although patients with early-stage disease have an excellent prognosis with a 5-year survival of more than 75–80%, there has been no effective therapy available for those with advanced disease, who are not eligible for transarterial chemoembolisation. We have reported earlier that 50% of HCC patients are diagnosed at an advanced stage (Greten *et al*, 2005). Recent advances in the development of targeted therapies have now also shown promising results. Here, we will discuss the results obtained using different molecular targeting agents and suggest new pathways for development of therapeutic options for HCC.

## Molecular carcinogenesis

More than 80% of all HCC occur in patients with liver cirrhosis. Chronic viral infections as well as alcoholic liver disease are the most common etiologies of liver cirrhosis and also represent the initiation point of hepatocarcinogenesis (El-Serag and Rudolph, 2007). Continuous cell death, which induces the release of different cytokines, as well as fibrogenesis and development of liver cirrhosis have been identified as early factors responsible for carcinogenesis. Integration of hepatitis B virus (HBV) DNA into the host genome not only induces chromosomal instability, but depending on the site of DNA integration may also activate oncogenes or inactivate tumour-suppressor genes. Moreover, viral proteins (HBx and preS) have oncogenic properties. HBx activates different promoter elements and triggers activation of transcription factors such as AP-1 and NF-*κ*B. Through this activity, genes, which are involved in cell cycle control, as well as apoptosis, are affected. A different molecular pathway has been suggested for hepatitis C virus (HCV)-induced hepatocarcinogenesis. In this case, HCV core protein affects cellular proliferation and apoptosis. In addition, chronic HCV infection can cause major immune responses, which might ultimately support hepatocarcinogenesis.

## Tumour angiogenesis

Hepatocellular carcinoma is a highly vascularised tumour, which makes vascular targeting approaches appealing for the treatment of HCC. Two studies using the endothelial marker CD34 to identify neovascularisation have reported that high microvessel density was a significant predictor to poor disease-free survival after resection (Ho *et al*, 2006; Wada *et al*, 2006; Poon *et al*, 2007). The mechanisms responsible for angiogenesis in HCC are not completely understood. However, one of the most important stimuli for the formation of new tumour vessels is the interaction of vascular endothelial growth factor (VEGF) with the VEGF receptors 1 and 2. Vascular endothelial growth factor promotes the growth, migration and morphogenesis of vascular endothelial cells and increases vascular permeability. Although well-differentiated HCC express higher levels of VEGF, it has also been shown that circulating VEGF can be detected in patients with more undifferentiated carcinomas (Torimura *et al*, 1998). Moreover, a correlation between VEGF expression and survival has been described (Poon *et al*, 2007). Other factors important for neoangiogenesis in HCC include fibroblast growth factor 2 (FGF2), angiogenin and angiopoietin.

## Growth receptors

**Figure 1:**
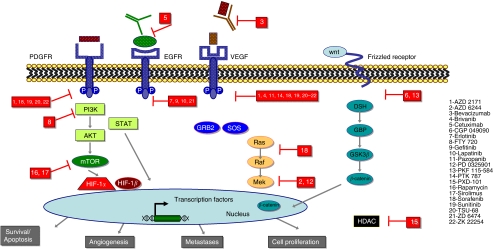
Schematic overview of key pathways in carcinogenesis and molecularly targeted therapy in hepatocellular carcinoma.

Tyrosine kinase receptors represent an attractive target for molecular therapy of HCC. The Ras–MAP kinase (MAPK) signal transduction pathway, as well as the phosphatidylinositol-3 (PI3K)–AKT kinase, are directly activated through the engagement of different growth factor receptors. A number of growth factor receptors are important in hepatocarcinogenesis such as the epidermal growth factor receptor (EGFR), the fibroblast growth factor receptor (FGFR), the hepatocyte growth factor receptor (HGFR), the stem cell growth factor receptor (c-kit), the platelet growth factor receptor (PDGFR) as well as the VEGF receptor. Engagement of these factors activates the Grb2/shc/SOS complex, which initiates the activation of the Ras–Raf–ERK1/2 MAPK signalling pathway and therefore ultimately leads to cell proliferation. A number of different small molecules, which are currently in clinical use or under early clinical or preclinical evaluation, block these pathways at different levels. There are a number of small molecules targeting TK activity of EGFR that are being evaluated for the treatment of HCC (Figure 1).

## Sorafenib for the treatment of advanced HCC

The multi-tyrosine kinase inhibitor sorafenib is the first and so far the only drug that has shown overall survival benefit in patients with HCC in a multi-centre, double-blind, placebo-controlled randomised phase III trial (SHARP trial). Median overall survival increased from 7.9 months in the placebo group to 10.7 months in the sorafenib group (hazard ratio in the sorafenib group, 0.69; 95% confidence interval, 0.55–0.87; *P*<0.001) (Llovet *et al*, 2008b). Sorafenib blocks the Ras–Raf kinase pathway in the tumour cell as well as the VEGF and PDGF receptor on the endothelial cells (Wilhelm *et al*, 2004). Although a number of studies suggest the relevance of the Ras–Raf pathway in primary liver tumours, surprisingly, only one study detected BRAF mutations in liver tumours. However, these tumours were primary bile duct tumours (Tannapfel *et al*, 2003) suggesting that a major mode of action in the treatment of HCC is the potent antiangiogenic potential of sorafenib. The most common grade 3 drug-related adverse events observed in the SHARP study included diarrhoea and hand-foot skin reaction, both of which occurred in 8% of all patients treated with sorafenib. Recently, a survival benefit has also been shown for Asian patients with advanced HCC treated with sorafenib. In this study, the median overall survival increased from 4.1 months in the placebo group to 6.2 months in the sorafenib group (hazard ratio in the sorafenib group 0.67; 95% confidence interval, 0.49–0.93; *P*<0.0155) (Cheng *et al*, 2008). Based on these two pivotal studies sorafenib has become the new standard of care for patients with advanced HCC. Future studies will define the role of sorafenib also in the adjuvant setting or in combination with transarterial chemoembolisation. Although until today no data is available on the combination of sorafenib with other molecular targeting agents for the treatment of HCC, one phase II study using the combination of sorafenib and doxorubicin has been performed. This study while showing good efficacy also revealed significant doxorubicin-related toxicities (Abou-Alfa *et al*, 2008) (Table 1).

## Results from phase I and II studies testing other approved targeting drugs

Sunitinib represents another small molecule that is currently being evaluated in a phase III trial for the treatment of HCC. Sunitinib is a multi-tyrosine kinase inhibitor that has already been approved for the treatment of renal cell carcinoma as well as gastrointestinal stroma tumours. It targets receptor tyrosine kinases of the split-kinase domain family such as VEGFR-1 and -2, PDGFR-α and PDGFR-β, c-kit and *FLT3* and the *RET* kinase (O’Farrell *et al*, 2003). A number of different preclinical models have shown an enhanced efficacy of tyrosine kinase inhibitors if the VEGF receptors on surrounding endothelial cells as well as the PDGF receptors on pericytes are blocked simultaneously. Results from different phase II studies in which patients with unresectable and metastasised HCC were treated with 37.5 mg sunitinib daily are shown in Table 1. This treatment was reasonably well tolerated and 9 out of 19 patients showed stable disease 12 weeks after initiation of treatment. A partial response was observed in one patient and a decrease in tumour permeability in a number of patients (Zhu *et al*, 2007a). In a second study in patients with unresectable HCC, patients were treated with 50 mg sunitinib daily. Although one patient showed a partial response in this study, the sunitinib dosage had to be reduced in 27% of the patients due to adverse effects. In five cases an acute decompensation of the liver cirrhosis was observed (Faivre *et al*, 2007).

Erlotinib at a dosage of 150 mg daily has been evaluated in two independent phase II trials in patients with advanced HCC. Although the objective tumour response rates observed were only minor (6% in one trial and no responses in the other trial), stable diseases were observed in 50 and 43% of the cases, respectively (Philip *et al*, 2005; Thomas *et al*, 2007a), with a PFS rate of 32 and 28% after 6 months and 24 weeks. Lapatinib, a different EGFR tyrosine kinase inhibitor, has shown promising results in a phase II trial with 2 out of 17 partial responses and a modest PFS of 2.3 months (Ramanathan *et al*, 2006) (Table 1). Moreover, gefitinib, an oral EGFR tyrosine kinase inhibitor has been tested in patients with advanced HCC as a single agent. However, the authors have concluded that gefitinib was not effective in patients with advanced HCC (O’Dwyer *et al*, 2006) (Table 1). Cetuximab, an EGFR blocking antibody, has been tested in multiple phase II trials. Zhu and colleagues reported a PFS of 1.4 months (Zhu *et al*, 2007b) and no objective responses. In our study we observed similar results with a TTP of 2 months (Grunwald *et al*, 2007) (Table 1).

Bevacizumab is a recombinant humanised antibody directed against VEGF. It has been approved in the United States by the FDA for the treatment of a number of different tumours including colorectal cancer, non-small-cell lung cancer and breast carcinoma. Partial responses have been observed in patients after bevacizumab monotherapy and disease stabilisation in 30% of treated patients has been documented (Siegel *et al*, 2008). Combination treatment of bevacizumab with EGFR targeting agents was shown to be safe and revealed promising initial results with a median time to progression of 9 months (Thomas *et al*, 2007b) (see Table 1). Overall, bevacizumab has shown promising results as a single agent as well as in combination with other targeted therapies and cytotoxic therapy (Zhu *et al*, 2006) supporting the idea of a vascular targeting therapy for the treatment of HCC.

## Reports from clinical trials testing new molecular targeting drugs for the treatment of HCC

AZD2171 is another small molecule, which by blocking the tyrosine kinase activity of VEGFR, PDGFR and c-kit, has shown remarkable effects in the treatment of glioblastoma. It has also been tested in a phase II trial in patients with non-resectable HCC. However, 84% of the patients experienced grade 3 toxicities at a dosage of 45 mg (Alberts *et al*, 2007). It remains an open question why AZD2171 was so poorly tolerated in patients with HCC. One possible explanation could be the underlying liver cirrhosis, which can impair drug metabolism significantly.

PTK787 is a compound that inhibits VEGFR1, VEGFR2, VEGFR3, PDGFR-β, Flt-3 and kit. It was tested in a phase III trial in patients with advanced colorectal cancer. However, the results from that trial were negative. In a murine HCC model oral administration of PTK787 induced tumour cell apoptosis and reduced vessel formation (Liu *et al*, 2005). Induction of hypoxia through ligation of the hepatic artery enhanced this effect (Yang *et al*, 2006). PTK787 might therefore represent an interesting combination treatment with transarterial chemoembolisation. The maximal tolerated dose was determined to be 750 mg daily in a phase I study of 18 patients with HCC and disease stabilisation was observed in 9 patients (Koch *et al*, 2005).

ZD6474 is a new oral inhibitor of VEGF receptors that additionally targets the EGFR/HER1 receptor. This drug has shown significant antitumour activity in preclinical tumour models and experimental data also suggests that it might inhibit metastasis formation. It is currently being tested in a phase II trial in patients with HCC. A phase I/II study of TSU-68, an oral angiogenesis inhibitor, which targets VEGF, *β-*FGF and PDGF, has shown good tolerability of this drug and preliminary data suggest its antitumour activity in patients with HCC. Brivanib, an oral VEGFR and FGFR tyrosine kinase inhibitor, is currently being tested in a phase I and II study in HCC patients and a phase III trial is being planned.

## Compounds in early development for the treatment of HCC

AZD6244 is a new MEK inhibitor, which is being evaluated for the treatment of HCC in preclinical models. The MEK inhibitor, PD0325901, which can be administered orally, has been evaluated in TGF-α transgenic mice in which liver cancers were induced by diethylnitrosamine treatment. PD0325901 led to a significantly smaller number of premalignant tumour cells in the treatment group. So far there is no data available on the effect of MEK inhibitors in patients with HCC.

The PI3K pathway is activated by a number of different growth factors and cytokines. PI3K activation leads to the expression of phosphoinositol triphosphate and activation of the AKT (PKB) kinase. AKT has multiple cellular targets, which suppress induction of apoptosis in the cell. One important substrate of the AKT kinase is the ‘mammalian target of rapamycin’ protein family (mTOR). The mTOR regulates the activation of p70 S6 kinase and the translational repressor proteins PHAS-1/4E-BP. These proteins control the cell cycle and have therefore an impact on cell proliferation. AKT activation is antagonised by the expression of the lipid phosphatase PTEN. In recent years, different molecules have been developed which target this pathway at various steps. The sphingosine receptor inhibitor FTY720 controls PI3K activity by inhibition of rac proteins. Treatment of HCC cells with FTY720 results in reduced cell motility, which might affect metastasis formation (Lee *et al*, 2005). The macrolid antibiotic rapamycin binds cytoplasmic FK506 binding protein (FKBP12) thereby inhibiting mTOR activity. This might be one reason why, sirolimus as well as its derivates block growth of tumours with an activated PI3K–AKT signal transduction cascade. The activity of PTEN is reduced in more than 50% of all HCC. In addition, hepatocyte specific knockout of PTEN promotes hepatocarcinogenesis in mice. Mammalian target of rapamycin inhibitors such as sirolimus suppress the proliferation of hepatoma cells *in vitro* as well as *in vivo* in a xenotransplant model. Interestingly, the survival benefit observed in mice correlated with a decreased vascularisation of tumours in mice. Different mTOR inhibitors such as temsirolimus and RAD001 are currently being evaluated in preclinical models of HCC and first phase I/II trials have been initiated.

A different targeting pathway includes Wnt proteins that function as ligands for the so-called Frizzled family of G-protein coupled receptors. β-catenin is activated by the Wnt signal transduction pathway and binds the transcription factor TCF (T-cell factor) to initiate expression of a number of genes, which are important for proliferation and cell survival including cyclin D1, c-myc and others. Disturbing the Wnt signal transduction cascade at different levels can cause a constitutive activation of this pathway, which promotes hepatocarcinogenesis. Indeed, activating mutations in the Wnt pathway have been observed in up to 40% of all HCC. It should be noted at this point that Wnt pathway also plays an important role in liver regeneration and proliferation of stem cells opening the possibility to potentially inhibit the proliferation of tumour stem cells. Although no molecules targeting this pathway have made their way into clinical evaluation, different drugs are currently in preclinical testing such as PKF115-584 and CGP049090.

Epigenetic modifications of the genome (mainly hypermethylation of CpG island and histone deacetylation) accumulate during hepatocarcinogenesis in chronically injured liver cells. It has been shown that a large number of tumour-suppressor genes are inactivated by epigenetic mechanisms in HCC. Success in epigenetic therapy (such as 5-aza-2-deoxycytidine and SAHA) has been achieved in both haematological malignancies and solid tumours. In HCC cell lines, chemosensitivity can be potentiated by epigenetic therapy. A multi-centre phase I/II trial on a novel histone deacetylase inhibitor, *PXD-101*, is currently underway in Hong Kong.

## Conclusion

In contrast to haematological malignancies such as CML, no single oncogenic event can be accused for the development of HCC. Instead, a multitude of different signalling pathways are affected in liver cancer cells making it difficult to focus molecular treatments. The results of recent clinical trials and the advent of the first systemic treatment for HCC point towards a multi-targeted therapeutic approach to this disease. The demonstration of an increase in overall survival of less than 3 months with the use of sorafenib in patients with advanced HCC can only be the beginning of a new era in the treatment of HCC. More drugs potentially targeting alternative pathways need to be evaluated in combination with sorafenib. In addition, more drugs targeting similar molecules need to be evaluated compared with sorafenib as recently suggested (Llovet *et al*, 2008a). However, understanding the exact mechanisms involved in hepatocarcinogenesis remains the fundamental condition for the development of new and more potential drugs for the treatment of HCC.

## Figures and Tables

**Table 1:** Summary of efficacy results obtained in recent phase II clinical trials using molecular therapies for the treatment of HCC in comparison to the results obtained with sorafenib in the SHARP phase III trial

**Regimen**	**No. of patients**	**RR %**	**Median PFS (P)/TTP (T), months**	**PFS at 6 months, %**	**Median survival, months**	**Reference**
Erlotinib	38	9	3.2 (P)	32	13	Philip *et al* (2005)
Erlotinib	40	0	3.1 (P)	28	6.3	Thomas *et al* (2007a)
Gefitinib	31	3	2.8 (P)	NR	6.5	O'Dwyer *et al* (2006)
Lapatinib	30	5	2.3 (P)	NR	6.2	Ramanathan *et al* (2006)
Cetuximab	30	0	1.36 (P)	3	9.6	Zhu *et al* (2007b)
Cetuximab	32	0	1.87 (T)	NR	NR	Grunwald *et al* (2007)
Cetuximab-GemOx	43	23	4.5 (P)	NR	9.2	Louafi *et al* (2007))
CapeOx+Cetux	25	33	4.3 (T)	NR	NR	O’Neil *et al* (2008)
Bevacizumab	46	13	6.9 (P)	65	12,4	Siegel *et al* (2008)
Bevacizumab	24	12.5	NR	NR	NR	Malka *et al* (2007)
GemOx+Beva	33	20	5.3 (P)	48	9.6	Zhu *et al* (2006)
CapeOx+Beva	30	10	5.4 (P)	40	NR	Sun *et al* (2007)
Cape+Beva	25	16	4.1 (P)	34	10.7	Hsu *et al*, (2007)
Beva+Erlotinib	34	21	9 (P)	75 (at 4 months)	19	Thomas *et al* (2007b)
Sunitinib	26	3.8	4.1 (P)	35	11.6	Zhu *et al* (2007a)
Sunitinib	37	2.7	5.2 (P)	35	11.2	Faivre *et al* (2007)
Sunitinib	23	6	NR	NR	NR	Hoda *et al* (2008)
Sorafenib+Doxo	47	4	8.6 (T)	NR	13.7 (6.5 for placebo)	Abou-Alfa *et al* (2008)
Sorafenib	300	2.3	5.5 (T)	62 (at 4 months)	10.7 (7.9 for placebo)	Llovet *et al* (2008b)

NR=not reported; PFS=progression free survival; RR=response rate; TTP=time to progression.
